# Real-time cell analysis system in cytotoxicity applications: Usefulness and comparison with tetrazolium salt assays

**DOI:** 10.1016/j.toxrep.2020.02.002

**Published:** 2020-02-07

**Authors:** Justyna Stefanowicz-Hajduk, J. Renata Ochocka

**Affiliations:** Department of Biology and Pharmaceutical Botany, Medical University of Gdańsk, Al. Hallera 107, 80-416, Gdańsk, Poland

**Keywords:** Cell index, Impedance, Microsensor electrodes, Tetrazolium salts, RTCA

## Abstract

Real-time cell analysis (RTCA) is a technique based on impedance and microsensor electrodes. RTCA system allows label-free, real-time, and continuous monitoring of cell adhesion, morphology, and rate of cell proliferation. The system offers a wide range of applications, mainly in toxicological studies, new drug screening, and microbiology. Here, we describe the usefulness of the system in different applications and compare this technology with conventional endpoint assays based on tetrazolium salts. We present advantages and disadvantages of the system and endpoint methods and their limitations in cytotoxicity investigations.

## Highlights

• RTCA system allows to easily monitor cell adhesion and proliferation.
• The real-time impedance technique is widely used in many toxicological studies.
• RTCA results are generally comparable with results from tetrazolium salts assays.
• RTCA analysis should be limited when drugs with electroactive additives are tested.
• Tetrazolium salts assays should be avoided when colored compounds are studied.

## Graphical abstract

**Figure:**
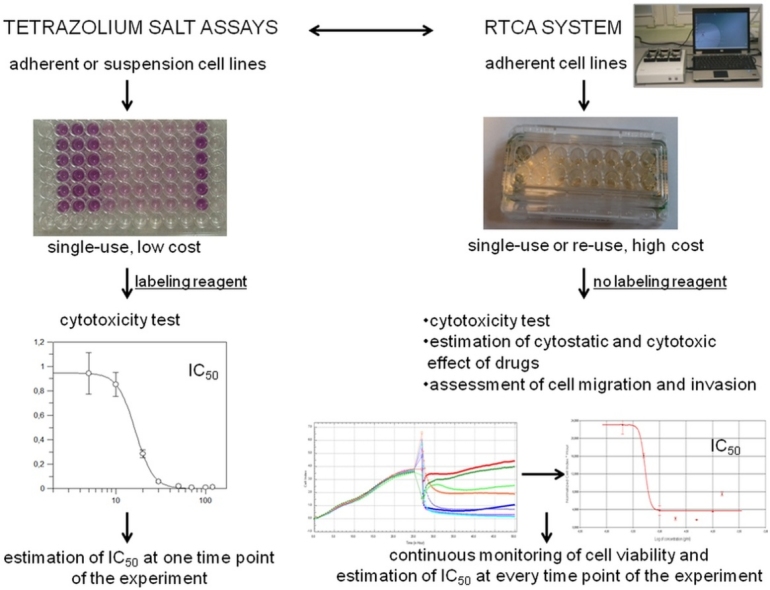

## 1 Introduction

**Fig. 1:**
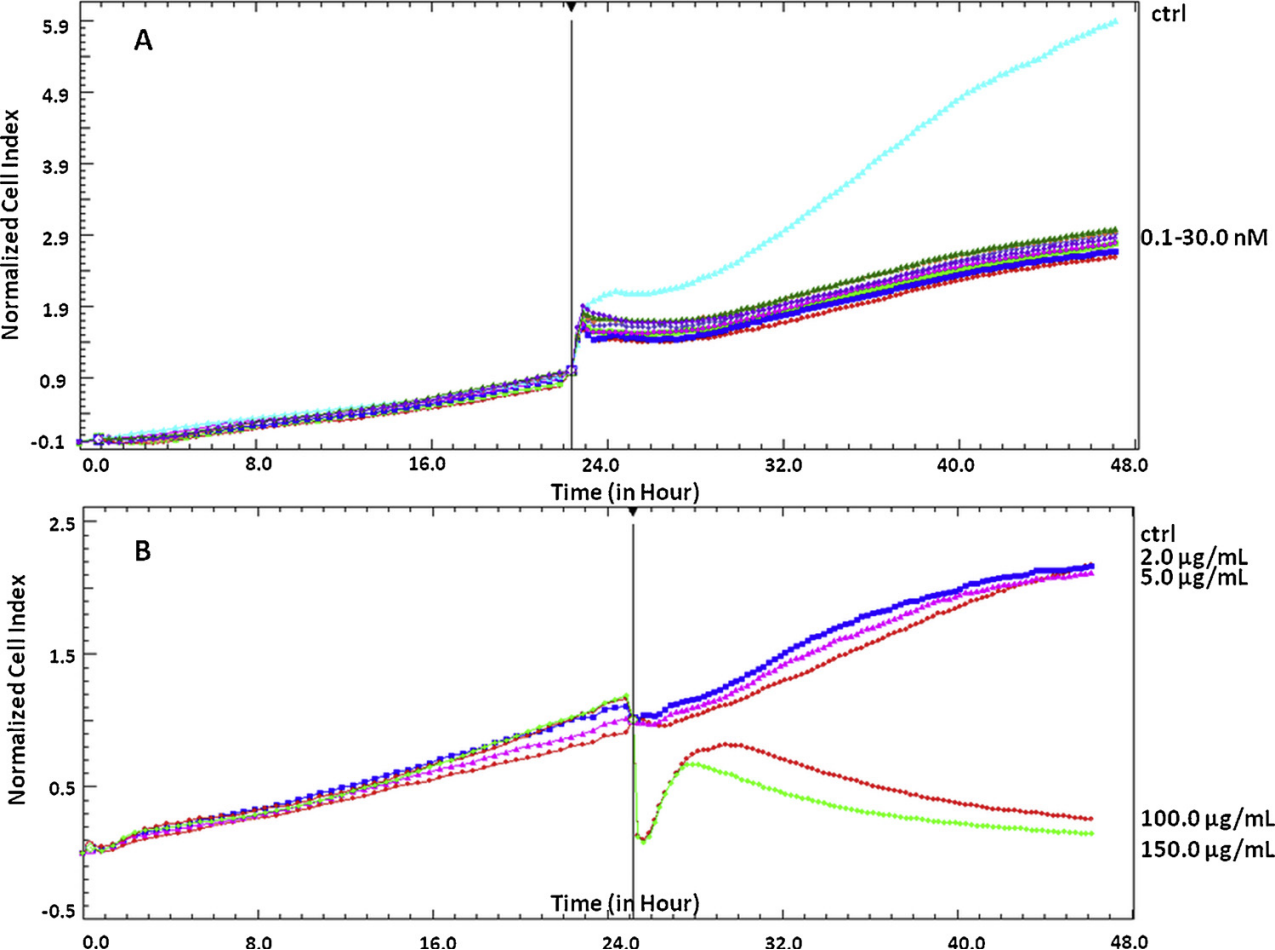
The effect of different compounds on HeLa cells in the RTCA system. The cytostatic activity is presented by vinblastine at concentrations of 0.1–30.0 nM (A); the cytotoxic activity is presented by fraction of *Kalanchoe daigremontiana* at concentrations of 2.0–150.0 μg/mL (B). The presented results come from the authors own research.

Real-time cell analyzer (RTCA) is a system that is based on electronic detection of biological processes. This system was used for the first time in 2008 as a more modern technique to real-time cell electronic sensing (RT-CES) system. Both systems allow label-free, real-time, and continuous monitoring of cellular adhesion, proliferation, growth, and morphology states [1]. The prototype of these chip-based techniques was an electrical biosensor – electrical cell-substrate impedance sensing (ECIS) system described by Giaever and Keese [2]. This device was equipped with small gold electrodes lying on the bottom of culture vessels. Similarly, today’s devices are also equipped with microelectrodes that cover the area of culture wells in plates of the systems. The passage of electrons and ions on sensor surfaces is affected by changes in properties of cells or molecules. In other words, electronic impedance of these sensor electrodes allows detecting the attachment of the cells on wells’ bottom and monitors their spreading at a particular time, expressed as the cell index (CI) value. This value is defined as (Z_i_ – Z_0_ Ω)/15 Ω, where Z_0_ is the background impedance of the well measured with medium alone and Zi is the impedance of the well measured at any time (t) with cells present. Thus, the CI is a reflection of overall cell number, adhesion quality, and cell morphology, which can change as a function of time [1]. Data are produced and collected during the whole time of running the protocol, and hence there is a possibility of assessing the effect of a tested compound on cells at any time of the experiment (Fig. 1).

The system generates proliferation profiles in one single experiment and enables to obtain IC_50_/EC_50_ values. In a study of new anticancer compounds, their profiles and data may be compared with profiles of well-known drugs, which trigger different cellular reactions [[3], [4], [5]]. This is useful in the selection of further methods for estimating compound action and helps to better understand mechanisms of toxicity as well as supports the selection of the best compound candidates in early drug development before entering animal testing [3].

Real-time and impedance technology offers a wide range of applications due to the availability of different kinds of RTCA systems. The basic system is called RTCA xCELLigence that uses 16-, 96-, or 384-well electronic microtiter plates. A version of the system – RTCA DP (dual purpose) analyzer – is used for measuring the kinetics of cell invasion and migration with electrically integrated Boyden chamber (CIM-Plate 16). One of a modern and the latest type of RTCA is RTCA iCELLigence instrument that is placed in a cell culture incubator, which transmits data wirelessly to control unit (iPad). The well sizes in plates of this system are larger than those in plates of xCELLigence analyzer, which enables the use of cells for complementary assays such as sequencing analyses, flow cytometry, western blotting, and imaging. The application of this system is similar to xCELLigence RTCA, and both systems are used in cell proliferation and differentiation study, cell- and compound-mediated cytotoxicity, receptor-mediated signaling, and quality control of cells [6]. Another version of the real-time system is xCELLigence RTCA Cardio that monitors cardiomyocyte contractility and viability in the presence of different drugs. This system enables the evaluation of cardiotoxicity for clinical safety and it is often used in research areas such as oncology drugs with short- and long-term toxicity, arrhythmia, and hypertrophy [[7], [8], [9]].

## 2 RTCA system in cytotoxicity investigations

The real-time cell analysis system has been already applied for several purposes and used in many experimental studies such as microbiological research [[10], [11], [12]], plant metabolites study [13,14], environmental toxicity [15,16], cellular function [1], and investigations of new potential anticancer drugs [17,18].

### 2.1 Plant extract and metabolites studies

Natural plant compounds are nowadays in focus of anticancer investigations due to the increasing interest on herbals as important agents in cancer treatment. Therefore, in this field RTCA system has been widely used both in study of whole plant extracts and isolated active compounds. Wang et al. explored the effect of soybean (*Glycine max*), a source of essential amino acids and flavonoids, on human breast cancer cell lines (MCF-7 and MDA-MB-231). The growth rate of the cells, measured by xCELLigence real-time cell analysis, was significantly inhibited in a dose-dependent manner [19]. Kayacan et al. used the xCELLigence system and studied the cytotoxic effect of methanol extract of *Centaurea nerimaniae* on human cervical cancer HeLa and MDA-MB-231 cells [20]. Harati et al. applied this system in the assessment of proliferation and viability of soft-tissue sarcoma cell lines after treatment with *Viscum album* extract. The results showed that this extract reduced viability of most of the tested cell lines [21].

The impedance technology has also been used in cytotoxicity studies of different plant metabolites. Many of them are focused on compounds from groups of glycosides (flavonoids, saponins, and alkaloids), which are widely distributed in plants.

Flavonoids are a large family of polyphenolic plant compounds. Quercetin, a well-known flavonoid, was studied in nasopharyngeal carcinoma cells. The results showed that this compound inhibited proliferation of the cells and also displayed synergistic effects on the cells in combination with cisplatin [22]. Braicu and Gherman investigated the antiproliferative effect of epigallocatechin gallate, a compound from subclass of flavan-3-ols, on triple-negative breast cancer cells Hs 578T. The results obtained by the RTCA analyzer that indicated reduction of cell proliferation were confirmed by 3-(4,5-dimethyl-2-thiazolyl)-2,5-diphenyl-2H-tetrazolium bromide) (MTT) test [17,23]. Apigenin and luteolin from flavones have antioxidant and antitumor effects. The activity of these compounds on cells was assessed in the breast cell line MCF-7 cultured in plates of the RTCA system to perform cell migration analysis [24]. Cardamonin – a compound from another subclass of flavonoids – chalcone was assessed for cardiotoxicity on cardiomyocytes. The results indicated that this compound did not inhibit contraction of the cells [13]. The flavonoid icariside II, isolated from *Herba Epimedii*, was incubated with melanoma cell lines (A375 and SK-MEL-5) and exhibited inhibitory effect on proliferation of these cells in a dose- and time-dependent manner [14].

Saponins have been also tested on cells *in vitro* by using the RTCA system, with estimation of their inhibition of cell proliferation and cytotoxic properties. In a study with two steroidal saponins isolated from *Paris quadrifolia,* strong cytotoxic activity was observed in HeLa cells [25]. Furthermore, RTCA proliferation profiles were useful in preliminary assessment of mechanisms of these compounds’ action in the cells. Ginsenoside (Rg1), a type of triterpene saponin and one of the active compounds in *Panax ginseng*, was tested in mouse cultured astrocytes *in vitro*. The results revealed that Rg1 was non-cytotoxic to astrocytes and also inhibited H_2_O_2_-induced apoptosis in the cells [26].

Next, from a group of alkaloids, chelidonine and homochelidonine from *Chelidonium majus* were tested in human lung adenocarcinoma A549 cells. The RTCA system was used to monitor cell adhesion, proliferation, and cytotoxicity after treatment with the metabolites. Both compounds showed antiproliferative activity; however, chelidonine was more active [27]. Moschamine, a type of an indole alkaloid occurring in *Centaurea* species, was tested in glioblastoma cell lines. The xCELLigence system and MTT assay were used for examining the viability and proliferation of the cells after treatment with the alkaloid [28]. In a study of other compounds, glycoalkaloids from *Solanum tuberosum* (α-chaconine and α-solanine), the RTCA system was used to monitor growth profile of RL95-2 estrogen receptor-positive human endometrial cancer cell line. In this study, the system was useful in estimating optimal cell density and the time for the compound addition to the cells in the experiment [29]. In another study with the analyzer, anticancer potential of Amaryllidaceae alkaloids was evaluated by screening with a panel of 17 different human cell lines. From 22 alkaloids, three of them (haemanthamine, lycorine and haemanthidine) exhibited significant cytotoxicity against all the tested lines, with IC_50_ values in the micromolar range. Furthermore, the RTCA system indicated that these compounds suppressed cell proliferation after 10 h of treatment [30]. xCELLigence analyzer was also appropriate for real-time screening of the effect of alkaloids obtained from *Rhizoma Coptidis* in HepG2 cells. In this study, cell growth inhibition and reduction of cell viability were observed [31]. Similarly, scoulerine, an isoquinoline alkaloid; a quindoline derivative; and securinine, a *Securinega*-type alkaloid were also tested in cancer cells with the RTCA system [[32], [33], [34], [35], [36], [37]].

Real-time monitoring system is also successfully used in estimation of herbal effects on non-cancer cells. For example, Kikowska et al. investigated the effect of callus extract of *Chaenomeles japonica* on viability, morphology, and proliferation of normal human skin fibroblasts. The results showed that the extract caused a significant increase in the proliferation rate of the cells in comparison to control cells, which indicated that this extract may be potentially used as a cosmetic ingredient on human skin [38]. In another study, plant extracts from *Syzygium aromaticum*, *Cinnamomum zeylanicum*, and *Salvia triloba* were tested in dental pulp stem cells using real-time monitoring [39]. The investigation revealed that these extracts could be used with biocomposites in dentistry as a promising osteogenic inducer and anti-inflammatory agent.

### 2.2 Drug screening

The RTCA system has been widely used in the study of both new and well-known cytostatic and cytotoxic drugs. Caltova and Cervinka determined the effect of selected cytostatics – cisplatin, paclitaxel, carboplatin, gemcitabine, topotecan, and etoposide – on the human ovarian cancer cell line A2780. This line showed different sensitivity toward the selected cytostatics, and the highest antiproliferative effect was associated with paclitaxel and topotecan [40]. Paclitaxel and cisplatin as nanotechnological drugs (nab-paclitaxel and liposomal cisplatin) were also studied in breast cancer cell lines with RTCA instrument [41]. Atienzar et al. evaluated impedance-based technology in pharmacology investigations by comparing RTCA profiles of different drugs such as calcium modulators, antimitotics, DNA damaging and nuclear receptor agents in human hepatocellular carcinoma (HepG2) cell line, as well as mouse neuroblastoma, cardiomyocytes and fibroblasts [42]. In this kind of study, the RTCA technology appeared to be a valid tool to assess drug effect on cells and discriminate cytostatic from cytotoxic activity. Furthermore, the authors highlighted that the RTCA proliferation profiles should help to better understand mechanisms of toxicity of tested drugs and select compounds for further *in vivo* study.

Real-time cell analysis is also used in studies that are based on exploration of better treatment of cancer diseases and limitation of side effects of drugs. For example, Kaya et al. tested motesanib, a small molecule inhibitor of vascular endothelial growth factor receptors that is administered orally. The xCELLigence system was used for testing motesanib alone and in combination with the selective DuP-697 COX-2 inhibitor in a human colorectal cancer cell line (HT29). The results showed that motesanib can be used in the treatment of colorectal tumors, and the combination with the inhibitor raises the possibility of using lower doses of the drug, therefore minimizing the side effects in the treatment [43]. Another colorectal carcinoma cell line (HCT116) was used to study the sensitivity of the cells to the anticancer drugs 5-fluorouracil and oxaliplatin [44]. In these cells, translationally controlled tumor protein (TCTP), an anti-apoptotic factor, was significantly overexpressed and the level of the protein increased in response to the drug treatment. However, when TCTP was knocked down in the cells, they were more sensitive to the tested drugs.

In the study of side effects of drugs, one of the most common effects is related with cardiotoxicity. Thus, many investigations have focused on this issue. Cardiotoxic side effects of drugs such as isoproterenol, carbachol, terfenadine, sotalol, ouabain, and doxorubicin were assessed with impedance technology using xCELLigence RTCA Cardio Instrument [33,45,34,35]. The system has interdigitated electrodes that detect the impedance changes of rhythmic contractions of cardiomyocytes [8]. These cells, isolated from pluripotent stem cells, were used in the study of cardiotoxicity profile of etoposide [9].

RTCA technology has been also applied in the assessment of safety of drug delivery systems – emulsions, liposomes, and lecithin dispersions [46]. This kind of investigation revealed that impedance technology is a useful tool for monitoring cell viability during treatment with drug systems, especially that some systems may interfere with reagents used in conventional assays [[47], [48], [49]].

Another useful application of RTCA system in cytotoxicity screening of compounds used in human treatment was reported by Urcan et al. [50]. The authors monitored the proliferation of human gingival fibroblasts (HGFs) treated with the most common components of dental resin materials: bisphenol-A-glycidylmethacrylate (BisGMA), hydroxyethylenemethacrylate (HEMA), triethyleneglycoldimethacrylate (TEGDMA), and urethanedimethacrylate (UDMA). The results indicated that HEMA was the least toxic among the tested monomers/comonomers. The study showed that the impedance system can be used as a rapid diagnostic tool in cell-based assays.

Most of reported studies with use of RTCA system describe toxicity tests on traditional immortalized cell lines *in vitro*. Recently, studies regarding the efficacy of molecular targeted drugs on tumor organoids and cells from patients [51,52] have also been described. Patient-derived tumor organoids (PDOs) are a preclinical model of cancer and are much better than traditional cell culture models due to their greater similarity to cancer diseases. Takahashi et al. evaluated *in vitro* different drugs such as: monoclonal antibodies, an anti-antibody-drug conjugate, and small-molecule inhibitors using lung PDOs [52]. In this study, the xCELLigence RTCA system was applied for testing the immune-checkpoint inhibitors – nivolumab and pembrolizumab on the lung cells in the presence or absence of peripheral blood mononuclear cells (PBMCs). The drugs caused cytolysis of the lung cells and induced death much stronger in the PBMC-mediated cells. These results showed that the system was suitable in selecting the most optimal therapy.

Other studies with RTCA system also showed the application of cells obtained from healthy patients or with cancers. These examples include malignant melanoma cells [53], patient – derived primary human breast cancer epithelial cells [54], ovarian cancer cells from a patient with serous ovarian cancer and endometrioid peritoneal cancer [55], chondrocytes [56], and mesenchymal stromal/stem cells [57,58]. In these papers, the impedance system was applied to establish culture conditions, monitor viability and migration of the cells, epithelial barrier function or cardiomyocyte beating. All these studies also confirm the usefulness of RTCA technology in a range of applications to patient samples [51].

### 2.3 Environmental toxicity investigations

Environmental toxicity studies are increasingly significant and widely conducted by many laboratories. In these investigations, both conventional cytotoxicity tests and RTCA technology are used. Leme et al. tested biodiesel and diesel blends in human T-cell leukemia (Jurkat) and human hepatocellular carcinoma (HepG2) cells and assessed their hazardous effects (xCELLigence system was used only in the case of adherent HepG2 line). Cytotoxicity was observed for waters contaminated with pure diesel and a blend with 5 % of biodiesel [15]. Otero-Gonzalez et al. tested a collection of inorganic nanomaterials in human bronchial epithelial cells (16HBE14o). The data obtained by the authors from RTCA analyzer were compared with results obtained by MTT assay [59]. Generally, there was a good correlation in cytotoxicity results between these two methods, which indicates that the RTCA technique is useful to rapidly screen nanoparticle (NP) toxicity. However, the authors observed significant differences in IC_50_ values from both assays for Al_2_O_3_ nanoparticles what can be caused by complex biological changes in the tested cells with the alumina nanomaterial, such as: subtle membrane altering effects or membrane depolarization detected by RTCA system but not by MTT assay [59].

In another study, a bronchial epithelial line and RTCA technology were used for the evaluation of the toxicity of arsenite (As(III)) adsorbed onto cerium dioxide (CeO_2_) NPs [60]. The results showed that this adsorbed As diminishes the inhibitory effect of As on the cells in comparison to As alone in aqueous solution.

In a study of toxic effects of pesticides that are approved as herbicidal products for commercial use, RTCA analyzer and MTT assay were applied. Three benzonitrile herbicides – bromoxynil, chloroxynil, and ioxynil – showed strong toxic effect in liver carcinoma HepG2 and kidney epithelial HEK 293T cells [16]. Furthermore, RTCA was also useful in evaluation of the toxicity risks of metabolic products formed by microorganisms from the tested herbicides in the environment.

Another environmental pollutant tested toward toxicity was dichlorodiphenyltrichloroethane (DDT) - widely used as an organochlorine pesticide for decades and is still present in environment due to its long-term persistence and low degradability [61]. The study of this compound was evaluated with positively and negatively charged nanomicelles on mouse fibroblast cells (NIH 3T3). The results obtained by the label-free real-time analysis clearly indicated that nanomicelles significantly decreased the toxic effect of DDT on the cells. This effect could be caused by reducing the amount of the pollutant in the culture medium due to its interactions with nanomicelles and/or detoxification during endocytosis of nanomicelle-DDT complexes in the cells [62]. Previous studies about DDT or other compounds and nanoparticles confirmed that this kind of complexes may change bioavailability and toxicity of pollutants [63,64]. One of these compounds is bisphenol A which is an endocrine disruptor. It is used in production of food containers, plastic bottles and other products useful in daily life. The compound is an environmental pollutant and is harmful for living organisms by induction of genotoxic effect - DNA double-strand breaks [65]. Furthermore, bisphenol A is an antagonist of an androgen receptor and generally affects male reproduction. The RTCA iCELLigence system was applied to analyze the effect of this compound on the mouse Sertoli cell TM4 viability [66]. The proliferation of the cells was inhibited after treatment with bisphenol A and this effect was caused by its anti-androgenic action. Other study performed also on the mouse cell line showed that a well-known pesticide – cypermethrin inhibited the proliferation of Sertoli cells and has the anti-androgenic effect [67].

RTCA analyzer was used in the testing of cytotoxicity of di(2-ethylhexyl)phthalate (DEHP) – a plasticizer and an endocrine disruptor which cause reproductive or developmental toxicity in humans. The inhibition effect on human embryonic stem cells (H9-hESCs) proliferation by DEHP was observed [68].

Black phosphorus (BP), which is used in industry, was also investigated and its toxicity was estimated on mouse fibroblast (NIH 3T3), human colonic epithelial (HCoEpiC), and human embryonic kidney cells (293T) [[33], [34], [35]]. This study revealed that toxicity of BP is size-dependent and the compound with the largest thickness and lateral size has the strongest toxicity. The results provided useful data for safe environmental and biomedical applications of black phosphorus especially that it is a promising compound in human cancer therapy.

### 2.4 Microbiological research

The technology based on impedance is widely used in virological and bacterial studies. One of these applications is quantitative measurement of infectious viruses that is based on virus-induced cytopathic effect (CPE). Conventional methods assessing virus replication are based on endpoint measurements, which are time consuming and do not provide information on the initiation of CPE. In comparison to these assays, the RTCA system monitors the changes in the virus propagation rate during the whole experiment [69]. For example, the system was used in the analysis of CPE of African swine fever virus [70], human enterovirus (HEV71) [71], West Nile virus, and St. Louis encephalitis virus [72]. Similarly, Piret et al. analyzed CPE of herpes simplex virus and human cytomegalovirus in Vero cells and human foreskin fibroblasts [73]. They also estimated concentrations of different antiviral drugs (acyclovir, foscarnet, ganciclovir) to reduce the effect of the pathogens in the cells. In other study, chikungunya virus and RTCA were used in the assessment of inhibitory activity of ribavirin on the pathogen replication [69].

Real-time analysis has also been successfully used in the detection of bacterial toxins. Jin et al. studied *Vibrio cholerae* toxin (CT) by monitoring CT-mediated cytotoxicity in four different cell lines – Y-1 mouse adrenal tumor cells, Chinese hamster ovary, small intestine epithelial cells, and mouse adrenal gland cells [11]. This study showed that the RTCA system may be applied for a rapid quantitative detection and identification of CT in comparison to conventional biochemical and immunological assays where CT detection is completed only after bacterial isolation. Similar research was done with *Clostridium difficile* [74]. In this study, the RTCA system was used for the detection of the bacterial toxins from stool.

The selected examples of the system application in toxicological, pharmacological, environmental and microbiological studies are presented in Table 1.

**Table 1:** Application examples of the real-time label-free RTCA system.

Category	Example	Cell line	Kind of study	Refs
**Natural compounds/** **extracts** extracts	*Glycine max*	human breast cancer cells MCF-7 and MDA-MB-231	cytotoxicity study	[19]
	*Centaurea nerimaniae*	human cervical cancer cells HeLa and breast cancer cells MDA-MB-231	cytotoxicity study	[20]
	*Viscum album*	sarcoma cells (fibrosarcoma HT1080, liposarcoma SW872, T778, MLS-402, synovial sarcoma SW982, SYO1, 1273, malignant fibrous histiocytoma U2197), primary human fibroblasts	cytotoxicity study	[21]
	*Chaenomeles japonica*	normal human fibroblasts	cytotoxicity study	[38]
	*Syzygium aromaticum, Cinnamonium zeylanicum, Salvia triloba*	human dental pulp stem cells	cytotoxicity study	[39]
plant metabolites *flavonoids*	quercetin	human nasopharyngeal carcinoma cells	cytotoxicity study	[22]
	epigallocatechin gallate	human triple-negative breast cancer cells Hs578T	cytotoxicity study	[17,23]
	apigenin, luteolin	human breast cancer cells MCF-7	cell migration analysis	[24]
	cardamonin	neonatal rat cardiomyocytes	cardiotoxicity study	[13]
	icariside II	human melanoma cells A375 and SK-MEL-5	cytotoxicity study	[14]
*saponins*	pennogenyl derivatives	human cervical cancer cells HeLa	cytotoxicity study	[25]
	ginsenoside Rg1	mouse cultured astrocytes	cytotoxicity study	[26]
*alkaloids*	chelidonine, homochelidonine	human lung adenocarcinoma A549	cytotoxicity study	[27]
	moschamine	human glioblastoma cells U251MG, T98 G, MRC5, HFL1	cytotoxicity study	[28]
	α-chaconine, α-solanine	human endometrial cells RL95-2	estimation of the optimal cell density and time for the compounds addition	[29]
	securinine	human cervical cancer cells HeLa	cytotoxicity study	[37]
	22 Amaryllidaceae alkaloids (e.g. haemanthamine, lycorine, haemanthidine)	17 different human cell lines	screening of cytotoxic activity	[30]
**Drugs** cytostatics	cisplatin, paclitaxel, carboplatin, gemcitabine, topotecan, etoposide	human ovarian cancer cells A2780	studies on antiproliferative effects	[40]
	cisplatin, paclitaxel as nanodrugs	human breast cancer cells MDA-MB-231, MCF-7	studies on antiproliferative effects	[41]
	carboplatin, cyclophosphamide, docetaxel, etoposide, 5-fluorouracil, idarubicin, irinotecan, vinblastine, vinorelbine, dasatinib, daunorubicin, doxorubicin, epirubicin, imatinib, sorafenib, sunitinib	human hepatocellular carcinoma cells HepG2, mouse neuroblastoma ND7/23, mouse cardiomyocytes and fibroblasts	comparison of RTCA results for different drugs, determination of RTCA conditions for different cellular models*	[42]
	5-fluorouracil, oxaliplatin	human colorectal carcinoma cells HCT116	studies on sensitivity of the modified cells to the drugs	[44]
	etoposide	human cardiomyocytes	cardiotoxicity study	[9]
	vinblastine sulfate	human cervical cancer cells HeLa, human breast cancer cells MCF-7	study on regeneration of RTCA plates	[36,]
antineoplastic	motesanib	human colorectal cancer cells HT29	study on limiting of the drug side effects	[43]
cardiac	isoproterenol, carbachol, terfenadine, sotalol, ouabain	iPS cell-derived cardiomyocytes (iPS-CMs) and primary cardiomyocytes	study on cardiotoxic side effects of the drugs	[45,[33], [34], [35]]
antiviral	acyclovir, foscarnet, ganciclovir	African green monkey kidney (Vero) cells, human fibroblasts	study on reducing the pathogenic viral effects in the cells	[73]
	ribavirin	African green monkey kidney (Vero) cells	study on reducing the pathogenic viral effects in the cells	[69]
immune-checkpoint inhibitors	nivolumab, pembrolizumab	patient-derived tumor organoids - lung cells with peripheral blood mononuclear cells (PBMCs)	cytotoxicity study	[52]
drug delivery systems	emulsions, liposomes, lecithin dispersions	normal human kidney embryonic cells HEK 293	cytotoxicity study	[46]
drug formulation additives	sorbitol, lactate, sodium hydroxide	human cervical cancer cells HeLa	limitations in the use of RTCA system*	[75]
**Pollutants** organic	biodiesel and diesel blends	human hepatocellular carcinoma cells HepG2	cytotoxicity study	[15]
inorganic	inorganic nanomaterials:Ag^0^, Al_2_O_3_, CeO_2_, Fe^0^, Fe_2_O_3_, HfO_2_, Mn_2_O_3_, SiO_2_, TiO_2_, ZnO, ZrO_2_	human bronchial epithelial cells (16HBE14o-)	cytotoxicity study	[59]
	As (III) adsorbed onto CeO2 nanoparticles	human bronchial epithelial cells (16HBE14o-)	cytotoxicity study	[60]
pesticides	bromoxynil, chloroxynil, ioxynil	human liver carcinoma cells HepG2, kidney epithelial cells HEK 293T	cytotoxicity study	[16]
	DDT	mouse fibroblasts	cytotoxicity study	[62]
endocrine disruptors/anti-androgenic effect	bisphenol A	mouse Sertoli cells TM4	cytotoxicity study	[66]
	cypermethrin	mouse Sertoli cells TM4	cytotoxicity study	[67]
	DEHP	human embryonic stem cells H9-hESCs	cytotoxicity study	[68]
**Microbiological studies** viruses	African swine fever virus	African green monkey kidney fibroblast-like cells COS-1	study on CPE	[70]
	human enterovirus HEV71	human rhabdomyosarcoma (RD) cells	study on CPE	[71]
	herpes simplex virus, human cytomegalovirus	African green monkey kidney (Vero) cells, human fibroblasts	study on CPE and antiviral activity of drugs	[73]
	chikungunya virus	African green monkey kidney (Vero) cells	study on CPE and antiviral activity of ribavirin	[69]
bacteria	*Vibrio cholera* toxin	mouse adrenal tumor cells, hamster ovary cells, intestine epithelial cells, mouse adrenal gland cells	monitoring of CT-mediated cytotoxicity	[11]
	*Clostridium difficile* toxins	human skin fibroblasts HS27	detection of the bacterial toxins from stool	[74]

* Studies showing limitations in the use of RTCA system.

## 3 RTCA system *versus* endpoint assays

### 3.1 Introduction

Toxicity endpoint tests are used mainly in the detection of the biological activity of tested compounds and can be used in different cell lines. These tests are based on basal or specialized functions of the cells that are connected with synthesis or release of specific molecules, activity of enzymes, alterations of metabolic pathways, or membrane functions [76]. One of the most common quantitative methods useful in cytotoxicity studies are tetrazolium reduction assays, which detect viable cells with active metabolism ranging from microbial origin to mammalian [77,78]. These assays are based on reaction with NADH or similar reducing molecules that directly or indirectly transfer electrons to the salts and reduce them to formazan products. Tetrazolium compounds used in the assays include MTT, 5-[3-(carboxymethoxy)phenyl]-3-(4,5-dimethyl-2-thiazolyl)-2-(4-sulfophenyl)-2H-tetrazolium inner salt (MTS), sodium 2,3-bis(2-methoxy-4-nitro-5-sulfophenyl)-5-[(phenylamino)-carbonyl]-2H-tetrazolium inner salt (XTT), and sodium 5-(2,4-disulfophenyl)-2-(4-iodophenyl)-3-(4-nitrophenyl)-2H-tetrazolium inner salt (WST-1) or sodium 2-(2-methoxy-4-nitrophenyl)-3-(4-nitrophenyl)-5-(2,4-disulfophenyl)-2H-tetrazolium salt (WST-8). They are widely popular in histochemistry, cell biology, biochemistry, and biotechnology academic labs as evidenced by a large number of published articles [75,77,79,80].

Endpoint assays are useful in testing both suspended and adherent cell lines, while the RTCA system is dedicated to adherent cells. However, Martinez-Serra et al. adapted the methodology based on the pre-coating of the cell culture surface with specific substrates and showed the possibility of using the system with leukemia/lymphoma suspension cells [81]. They also compared the cytotoxicity results obtained in the MTT assay and RTCA and showed that both data were similar in the two methods.

Protocols with testing of non-adherent cells by RTCA system describe covering the plates with fibronectin, laminin, collagen or gelatin [81,82]. Also, selected antibodies may be used to immobilize liquid cells (*e.g.* K562, Raji, MEC2 or Daudi) on the plate bottom, what is recommended by the RTCA manufacturer [83]. The coating substrates can also be used in experiments with adherent cells to improve their attachment to RTCA plates [42]. However, coating conditions should be selected and optimized depending on a cell line due to the fact that they have influence on cell adhesion and spreading, and eventually on RTCA results.

### 3.2 Advantages and disadvantages of real-time monitoring of cells and endpoint tetrazolium salt assays

The endpoint assays are rapid, simple, relatively inexpensive methods to screen a large number of compounds over a wide range of concentrations. The results are apparent visually, which is very useful if rapid qualitative results are required [84]. Despite of these advantages, there are a lot of problems with the assays. One of the major disadvantages is optimization of whole experiment at various time points to choose the best point for final reading. Many cell lines could proliferate after selecting of too short endpoint time [85]. Additionally, toxic substances in low concentrations can stimulate cell activity. That is why in some experiments the value of IC_50_ for a 24-hr incubation period is totally different from 48-hr incubation. This makes the endpoint-dependent toxicity much less reliable than generally anticipated [86]. In the tetrazolium salts methods, the obtained results show cytotoxicity only in one time point and there is no possibility to see any cellular changes between time points during an experiment.

The impedance-based technology enables to measure an entire experiment through many time points and creates curves that reflect cellular proliferation, growth, and morphological changes before and after adding a compound. The obtained data enable to calculate IC_50_ as well as EC_50_ in every time point of the experiment. This is why the system is very useful in screening new potential cytotoxic drugs since it overall shows the effect of a compound on cells in real time [1,3].

**Fig. 2:**
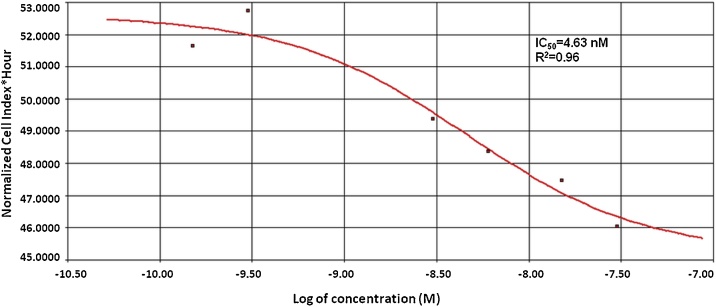
Calculation of IC_50_ value of vinblastine in HeLa cells based on sigmoidal dose response and area under curve in a time period *vs* concentration. The presented results come from the authors own research. R^2^ - the coefficient of determination.

For calculating IC_50_/EC_50_ value, the RTCA software uses the sigmoidal dose-response equation to apply curve fitting to the experimental data points [5]. A few curve types are generated in the system and present CI values at a time point/period *versus* compound concentrations (Fig. 2).

While endpoint assays can only provide a single IC_50_ value, the calculation of the time-dependent IC_50_ is one of the important features of RTCA, allowing the user to determine and derive the kinetic dependency parameter for compound effectiveness and potency [4]. The second type of curves generated by the system – the dose response curves – reflects the dependency of the CI value on the compound concentration. The choice of the curve type for analyzing data depends on the type and condition assay such as cytotoxicity, receptor activation, or cell adhesion.

A significant disadvantage of the tetrazolium salt assays is the fact that formazan products are sometimes reduced by the chemicals or materials that are present or generated in the culture media during the assay. Substances with intrinsic reductive potential reduce tetrazolium reagent and therefore may give false-positive results [[47], [48], [49]]. One of well-known compounds interfering with tetrazolium salts are antioxidants. Bruggisser et al. tested selected flavonoids (kaempferol, resveratrol), vitamins (ascorbic acids, vitamin E), and plant extracts (*Hypericum perforatum* and *Cimicifuga racemosa*). They observed that all these agents led to direct reductive potential in a cell-free system. Furthermore, when cells treated with kaempferol were washed before addition of MTT tetrazolium, the reduction of the dye was reduced significantly [47]. Other authors also showed interactions between different compounds and tetrazolium salt. Interference has been reported with whole serum and serum proteins [49,87,88], immunoglobulin, and heparin [49]. Also, lipids or liposome particles may interfere with tetrazolium salt and final results may be affected [89]. Thus, it is extremely important to perform a cell-independent chemical reaction before every experiment with tetrazolium salt and tested compounds.

Some substances may also inhibit the activities of different enzymes such as dehydrogenases and therefore it is important to consider what effect is expected and what cell death mechanism is predicted in these assays [90]. On the other hand, many non-mitochondrial dehydrogenases and flavin oxidases are able to reduce tetrazolium salt [91,92]. It has been shown that cells with inactivated mitochondria can also produce the same amount of formazan compared to cells with operative mitochondria [85]. However, for most viable cells, mitochondrial activity is constant and thereby an increase or decrease in the number of viable cells is linearly related to mitochondrial activity.

In MTT assay, cellular reduction of MTT yields an aqueous insoluble formazan, with visible crystals contained both in the surrounding medium and within the cells. For this reason, removal of the culture medium and addition of a solvent are required to dissolve and disperse the formazan to generate maximum absorbance [93]. The step of removal of medium should be carried out with care due to risk of dislodging of formazan crystals especially if an aspirator is used. Furthermore, color of the residual medium with phenol red may interfere with the color of formazan solution and this can also produce false-positive results. Recently, the latest and more convenient modifications of MTT test have been described (MTS, XTT, and WST assays). In these assays, tetrazolium reagents are reduced to generate formazan products that are directly soluble in cell culture medium, which makes the protocols easier [78]. However, false-positive results may also be generated in these tests due to interactions between the formazan product and an investigated compound. Colorimetric assays based on tetrazolium salts should not be used in testing of colored compounds/drugs, especially with less pronounced therapeutic effects. Cai et al. confirmed this by testing curcumin on HeLa and A549 cell lines [75]. The IC_50_ values obtained by CCK-8 assay significantly differed from the values in RTCA experiments. On the other hand, the cytotoxicity of doxorubicin hydrochloride (which is colored, but it has strong pharmaceutical effect) was similar in both CCK-8 assay and RTCA analysis. To obtain toxic effect in the cells, less amount of doxorubicin was used which resulted in a lack of significant differences in the IC_50_ results.

Finally, in all the end-point assays result of absorbance measurement is dependent not only on type and color of a tested compound but also on tetrazolium salt concentration added to cells as well as on cell density because they directly impact on the amount of produced formazan [93].

The xCELLigence system does not have these disadvantages. The technology enables accurate real-time monitoring of cell behavior without any need to manipulate the medium during the experiment or addition of any labels, which may alter cell function. Despite the fact that the system is label free, fluorescent labeling can be done during the experiment or cells can be stained post-experiment. Furthermore, cells could be harvested from the plates for subsequent analysis [1].

**Fig. 3:**
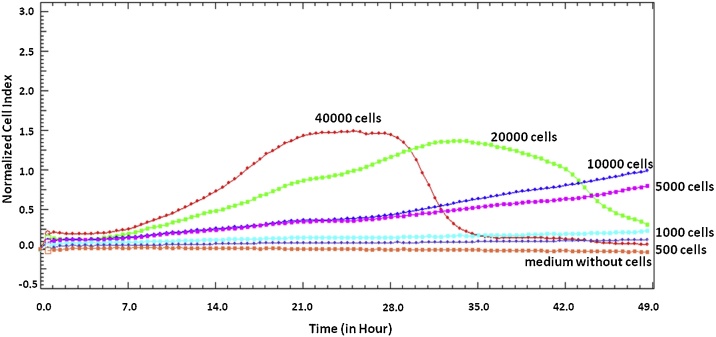
The RTCA proliferation curves of HeLa cells in different densities. The presented results come from the authors own research.

Density of cells is an important factor in both endpoint assays and the RTCA system. First of all, in both methods it is required to determine the optimal cell count for every cell line. In endpoint tests, the absorbance value of control cells without a tested compound should be between 0.75 and 1.25. Then, both stimulation and inhibition of cell proliferation can be measured after addition of a drug. In the RTCA system, the manufacturer recommends reaching a CI of 1 or higher, with a minimum CI of at least 0.5 (Fig. 3) [42,94].

Determination of cell numbers is a crucial step in studies focused especially on cytokinetics and cell toxicity [95]. The concentration of cell seeding in plates and time between seeding and adding a compound to cells may be different for every cell line. The wide range of cell lines and compounds that can be tested with the system makes it difficult to design a universal protocol suitable for each type of cells. A few protocols for different cell lines have been optimized and described recently [10,42]. Furthermore, in the case of cells that are not strongly adherent to the substrate and are easily to detach, even in small differences of temperatures in the environment, cytotoxicity results from the RTCA system should be confirmed by other methods. This is due to the high sensitivity of the impedance technology and presence of microelectrodes in the RTCA plates. This feature of the system may result in detection of toxicity effects of tested drugs with higher sensitivity in comparison to tetrazolium salts assays [40,46]. Moreover, Atienzar et al. pointed out that a decrease in cell index values is not always associated with cell death effect and may be caused by rapid changes in cellular morphology, size or attachment to RTCA plates [42].

The application of RTCA system in cytotoxicity tests should also be considered when drugs containing additives such as: sorbitol, lactate, and sodium hydroxide are studied [75]. These compounds from drug formulation may alter the impedance due to their dielectric properties. Therefore, RTCA system is suitable in testing of pure compounds or drugs without any electroactive components.

Despite the disadvantages of the endpoint assays and RTCA system, both methods are reliable and suitable in cytotoxicity investigations, and they generate mostly comparable results. Braicu and Gherman investigated the effect of epigallocatechin gallate in triple-negative breast cancer cells (Hs578 T). The dynamic real-time monitoring of the cells treated with the compound confirmed the antiproliferative effects obtained by MTT test [23]. Vistejnova et al. examined cell proliferation of 3T3 mouse fibroblasts, HaCaT keratinocytes, normal human epidermal keratinocytes (NHEK), and normal human dermal fibroblasts. Only for NHEK cell line, the results obtained from the xCELLigence system were not comparable with those from MTT assay. This could be accounted to the specific morphological characteristics of NHEK cells [95]. Many other authors compared the impedance-based method with tetrazolium salt assays [18,21,22,40,59,69,81].

In spite of more and more use of the RTCA technology, the main limitation of this system is the cost of experiments. E-plates are expensive, single use, and disposable. However, some authors indicate that E-plates can be regenerated and reused several times without significantly affecting experimental results [36,]. Other studies also show that gold microelectrodes can be used more than one time [96,97]. The regeneration process includes trypsin digestion, rinsing with ethanol and water, and a spinning step. Previously studies on regeneration of metal chips (gold or silver/aluminum/nickel) were done with regarding to the surface plasmon resonance (SPR) technique that enables label-free and in real-time detection of biomolecular interactions [98,99]. All these protocols show that the regenerated chips can be applied to obtain repeatable and reproducible results.

The characteristics of the RTCA technology and endpoint assays are presented in Table 2.

**Table 2:** The comparison of tetrazolium salt assays and RTCA system.

	Cytotoxicity assays	
	Tetrazolium salt assays	RTCA system
Kind of cells	Adherent and suspension	Mostly adherent (suspension cell lines after pre-coating of the plate bottom)
Monitoring of cells	Under a microscope at selected time points	Real time and continuous
Obtain results	At one selected time point	At every time point of the experiment
Labeling reagent	Tetrazolium salts	No reagents
Detection principles	Spectrophotometric measuring absorbance of formazan solution produced by living cells with active dehydrogenases	Recording the electrical impedance from the arrayed gold microchips covered the bottom of RTCA plates. Changes in impedance values reflect changes in cellular physiology and proliferation. CI - the basic obtained parameter
Contamination with assay reagent	Yes	No
Use of cells after experiment	No possibility to use	Use of cells for other assays (western blotting, imaging, *etc*.)
Optimization of cell density prior to experiment	Required	Required
Cost	Low cost	Costly
Use of plates	Disposable and single use	Disposable and single use, but possible regeneration and re-use
Applications	• Cytotoxicity test of natural and synthetic compounds, drugs, plant extracts	• Monitoring of cell adhesion, viability, and proliferation rate • Cytotoxicity test of natural and synthetic compounds, drugs, plant extracts • Assessment of cell migration and invasion • Measuring of cardiomyocyte contractility and viability • Differentiating cytostatic from cytotoxic effect of drugs • RTCA proliferation profiles are useful in study of mechanism action of new drugs by comparison with profiles of well-known drugs
Limitations of application with	• Colored compounds • Compounds that interfere with tetrazolium reagents (e.g. antioxidants)	• Drugs with electroactive additives • Less adherent and suspension cell lines

## 4 Conclusions

The real-time cell analysis system is a modern and useful technique applied in many areas of research. Despite high costs of experiments and some limitations, the system provides a high throughput and quantitative method for continuous and real-time monitoring of cell proliferation and cellular morphological changes. The system is used in pharmacological, toxicological, and microbiological studies, mainly to evaluate cytotoxicity of new drugs and improve the effects of different therapies. However, it is worth emphasizing that classical endpoint assays, due to low cost and simplicity, will still be widely applied as basic methods in many *in vitro* studies, especially in the need to confirm the RTCA results.

## Declaration of Competing Interest

The authors declare that there is no conflict of interest regarding the publication of this paper.
